# Abundance of megalin and Dab2 is reduced in syncytiotrophoblast during placental malaria, which may contribute to low birth weight

**DOI:** 10.1038/srep24508

**Published:** 2016-04-13

**Authors:** Jared Lybbert, Justin Gullingsrud, Olga Chesnokov, Eleanor Turyakira, Mehul Dhorda, Philippe J. Guerin, Patrice Piola, Atis Muehlenbachs, Andrew V. Oleinikov

**Affiliations:** 1 Charles E. Schmidt College of Medicine, Florida Atlantic University, Boca Raton, FL, USA; 2 Seattle Biomedical Research Institute, Seattle, WA, USA; 3 Mbarara University of Science and Technology, Mbarara, Uganda; 4 Centre for Tropical Medicine and Global health, Nuffield Department of Clinical Medicine, University of Oxford, Oxford, UK; 5 Epicentre, Mbarara, Uganda; 6 University of Washington, Seattle, WA, USA

## Abstract

Placental malaria caused by *Plasmodium falciparum* contributes to ~200,000 child deaths annually, mainly due to low birth weight (LBW). Parasitized erythrocyte sequestration and consequent inflammation in the placenta are common attributes of placental malaria. The precise molecular details of placental changes leading to LBW are still poorly understood. We hypothesized that placental malaria may disturb maternofetal exchange of vitamins, lipids, and hormones mediated by the multi-ligand (n ~ 50) scavenging/signaling receptor megalin, which is abundantly expressed in placenta but was not previously analyzed in pregnancy outcomes. We studied abundance of megalin and its intracellular adaptor protein Dab2 by immunofluorescence microscopy in placental biopsies from Ugandan women with (n = 8) and without (n = 20) active placental malaria. We found that: (a) abundances of both megalin (p = 0.01) and Dab2 (p = 0.006) were significantly reduced in brush border of syncytiotrophoblast of infected placentas; (b) amounts of megalin and Dab2 were strongly correlated (Spearman’s r = 0.53, p = 0.003); (c) abundances of megalin and Dab2 (p = 0.046) were reduced in infected placentas from women with LBW deliveries. This study provides first evidence that placental malaria infection is associated with reduced abundance of megalin transport/signaling system and indicate that these changes may contribute to the pathology of LBW.

During *P. falciparum* infection, pregnant women can suffer from placental malaria (PM), where parasitized erythrocytes (PE) sequester in the placenta^1^, which can lead to inflammatory response. PM contributes to about 200,000 neonatal and 10,000 maternal deaths annually in malaria endemic regions^2^. Infant mortality in PM is largely due to low birth weight (<2.5 kg), in addition to stillbirth, and abortion^3^. Sequestration of PE in the placenta occurs at the boundary surface of the syncytiotrophoblast^4^ – the interface between maternal blood and fetal vasculature where maternofetal exchange takes place^5^. Anatomically, fetal blood vessels branch in the placenta into villi that are covered by a single layer of multinucleated cell called the syncytiotrophoblast, which is created by fusion of underlying cytotrophoblast cells. The apical surface of the syncytiotrophoblast has microvilli (brush-border) expanding its surface to ~12.5 m^2^ for extensive molecular exchange^5^. Sequestration of PE on this surface can lead to macrophage infiltration into the intervillous space, local inflammation, and pathological changes in the syncytiotrophoblast^4^,^6^. These processes, in turn, may lead to reduced maternofetal exchange and reduced fetal growth during gestation, and finally to LBW and other poor outcomes including premature birth, pre-eclampsia, and small for gestational age babies^7^,^8^,^9^. Recent insights into mechanisms of the placental pathological processes during PM suggest involvement of various pathways, including angiogenesis^10^,^11^, insulin-like growth factor (IGF-1) axis^12^, and, potentially, mammalian target for rapamycin (mTOR) (all extensively reviewed in refs 6,13). Nevertheless, the molecular details of these processes, as well as the involvement and role of other proteins and pathways, are still poorly understood.

In this respect the glycoprotein megalin (also called gp330, gp600, and LRP2) is a particularly interesting candidate. It is a large (~600 kDa) multi-ligand single-spanning trans-membrane endocytic receptor with substantial physiological functions. It belongs to an ancient^14^ low density lipoprotein receptor family. The importance of this receptor has been demonstrated in a large number of experiments across multiple organs^15^, though its role in the placenta is not yet well characterized. It has been shown that megalin expresses in placenta and is localized on the surface of the syncytiotrophoblast^16^,^17^. Levels of megalin expression in the placenta are third highest following its expression by thyroid and kidney proximal tubular cells^18^. Many functions of megalin have been studied in kidney and in early embryonic development^15^,^19^. In mice, megalin expresses at very early stages of embryonic development^20^. Knockout of the megalin gene in mice leads to perinatal death (only 2% newborn mice survive) and severe pathologies in various organs, most notably in brain morphology, as well as in the kidney and lung^20^,^21^. Also, megalin-deficient mouse fetuses at mid-gestation were significantly smaller than wild-type^20^. It was hypothesized that developmental deficiency in these megalin knockout animals might be explained, at least in part, by a vitamin and lipoprotein deficiency due to defective/insufficient transport of these molecules (see below) to the fetus through megalin endocytosis in the yolk sac and placenta^19^,^20^. In humans, placental cytotrophoblast and syncytiotrophoblast megalin starts to express at least as early as 7–8 weeks gestation^22^ and expresses through term^16^,^17^,^22^,^23^. Substantial expression of megalin in the syncytiotrophoblast brush border^16^,^17^ indicates its importance for placenta function. In humans, mutation preventing megalin expression are extremely rare and only about a dozen surviving patients with Donnai-Barrow syndrome have been previously described^24^, with pathologies similar to those observed in megalin-knockout mice.

In epithelial cells, megalin is transferred with its cargo to the base of microvilli and is internalized through coated-pit endocytosis. We have demonstrated earlier that megalin interacts with a phosphotyrosine-interacting domain of the adaptor protein Dab2^25^,^26^, which binds to the internalization motif^27^ in the megalin cytoplasmic domain. Dab2 is expressed in various tissues including syncytiotrophoblast and in trophoblast cells^28^,^29^. Its mRNA expression in placenta is highest among all tissues^18^. Based on previous studies of others on Dab2 functions^28^,^30^,^31^, we hypothesized that megalin and Dab2 might be involved in both signal transduction as well as in endocytosis through their interactions^25^. This hypothesis was experimentally confirmed in multiple studies^15^,^19^,^32^,^33^. As Dab2 is important for cell growth suppression^28^,^30^, it may also have an important role in placental growth and development.

Megalin interacts with an enormous suite of about 50 extracellular ligands which belong to several unrelated groups of proteins^15^. These ligands include nutrients, hormones and their carrier proteins, vitamin-binding and signaling molecules, morphogens, and extracellular matrix proteins including serum proteases and their inhibitors. Internalized molecules are either transcytosed or released in endosomes or lysosomes (vitamins, cholesterol, etc.) and then can be utilized within the cell or excreted on the cell surface by various transporters. Clearly, such diverse functions might have significant impact on the maternofetal exchange of nutrients and signals, as well as affect the homeostasis of various important molecules. This, in turn, may lead to placental pathology resulting in poor outcome for fetal development, including LBW, if the megalin system is somehow disturbed.

A few interesting observations suggest that the megalin system might be affected in PM. When the megalin gene is knocked out, the number of microvilli and the amount of endocytosis are significantly reduced in kidney^21^. These changes are reminiscent of loss of syncytiotrophoblast microvilli reported during PM^4^ and strongly support the idea that megalin expression/distribution in PM might be disturbed. In addition, inflammatory processes, similar to that observed during Heymann nephritis, can lead to shedding of the megalin exodomain^34^.

To get insights into the megalin system during PM, we determined the abundance of megalin and its intracellular adaptor protein Dab2 in formalin-fixed paraffin-embedded placental samples obtained from women living in malaria endemic areas of Uganda, with and without PM, who had normal and low birth weights of newborn babies (Table 1 and Supplementary Table 1).

## Results

### Patient characteristics

Samples were selected based on availability of samples from previous study^35^,^36^. Summary clinical information is presented in Table 1, and data from individual samples are presented in Supplementary Table S1. Groups of Ugandan mothers without (PE−) and with (PE + ) placental infection at delivery were similar in respect to parity, hemoglobin levels, estimated gestational age, and birthweight of newborns.

### Expression of megalin and Dab2 in human syncytiotrophoblast in term placenta

Immunofluorescence studies confirmed substantial expression of megalin in the syncytiotrophoblast of human term placenta as previously reported^17^,^22^. However, this expression was not uniform throughout the syncytiotrophoblast; parts of the syncytiotrophoblast brush border may express very little of megalin (and Dab2), if any, as was noted previously for megalin in placenta^22^. Therefore, quantitative comparison of protein abundances in various samples is challenging. To overcome this difficulty the following steps were taken: (1) protein abundances were measured in the parts of syncytiotrophoblast brush border (BB) with the highest signal as reflecting the ability of the tissue to express these proteins and (2) repeated blind experiments were performed using different antibody preparations. Three non-overlapping areas encompassing the syncytiotrophoblast BB from each placental section were selected for the highest density of signal, measured, and averaged, as described in the Methods. Supplementary Figure S1 demonstrates that measurements obtained in independent blind experiments, using two independent preparations of anti-megalin C-terminal peptide (in cytoplasmic tail) antibodies and two preparations of anti-Dab2 antibodies raised against different regions, strongly correlate, suggesting that our measurements accurately reflect the levels of abundance of these proteins. As anti-megalin prep 2 (more recent preparation) antibody and anti-Dab2 commercial antibody had higher signal according to linear regression analysis (Supplementary Figure S1), they were used for all analyses described below.

### Placental malaria is associated with reduced abundance of megalin and Dab2

**Figure 1:**
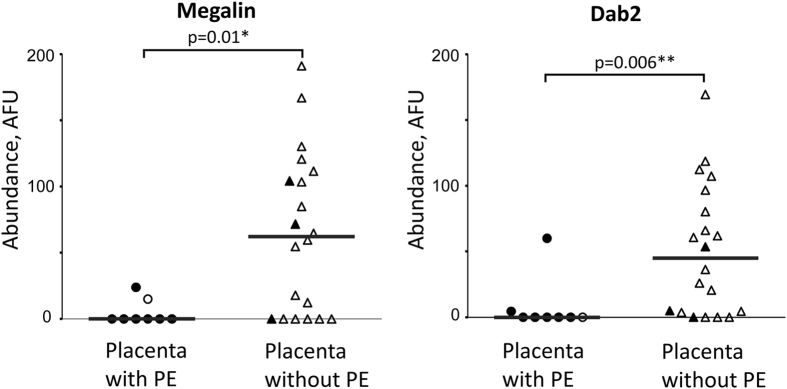
Megalin and Dab2 abundance is reduced in brush border area of syncytiotrophoblast in placentas with malarial infection at delivery. Abundance of megalin and Dab2 was assessed in brush border of placentas with PE (n = 8) and without PE (n = 20) using immunofluorescence assay as described in Methods. Medians of arbitrary fluorescence units (AFU) are reported (gray bars). Filled symbols represent samples with extracellular hemozoin in fibrin indicative of previous (resolved) placental infections, clear symbols represent samples without hemozoin. Protein abundance between 2 groups was compared using Mann-Whitney test. PE – parasitized erythrocytes in the placenta at the time of delivery.

The abundance of both megalin and Dab2 was reduced in the syncytiotrophoblast brush border in malaria-infected placentas by indirect fluorescence microscopy, with statistical significance for both proteins (Fig. 1). Data on parity is available for 22 samples used in this analysis (Supplementary Table S1). Only including these 22 samples in the analysis shows that median parity is not different (p = 0.88) for groups stratified by placental infection, while abundance of both proteins is still reduced in the infected group (Supplementary Figure S2), demonstrating that parity is not a confounding factor. Samples with previous (resolved) malaria infection, as defined by the presence of extracellular hemozoin (Hz) in fibrin, are indicated in Fig. 1. If samples with active and past placental infection were combined, megalin and Dab2 continue to demonstrate a statistically significant reduction of abundance in these samples against normal controls (p = 0.04 and 0.005 for megalin and Dab2, respectively). Two data points for megalin and one for Dab2 with past (resolved) placental infections were above the median protein abundance value in the group of non-infected placenta samples (Fig. 1), which may point out the possibility for restoration of megalin and Dab2 expression in the placenta after placental infection is cleared. Similar results (as in Fig. 1) were obtained when protein abundance was measured in the entire syncytiotrophoblast when cytoplasmic staining was included (though fluorescence values were lower).

**Figure 2:**
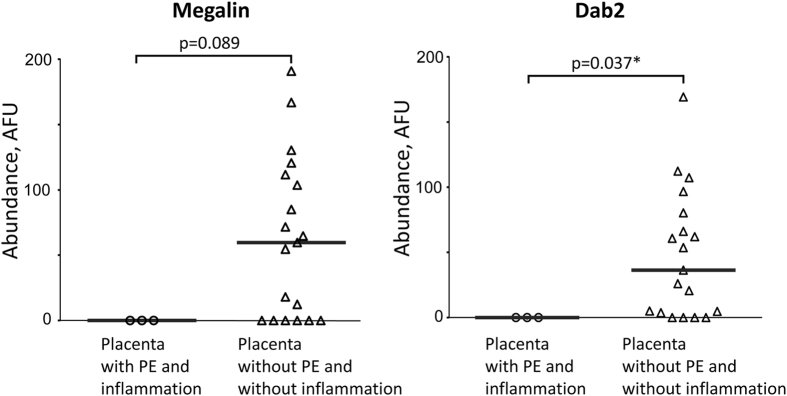
Megalin and Dab2 abundance is reduced in brush border area of syncytiotrophoblast in placentas with malarial intervillous inflammatory infiltrates. Megalin and Dab 2 abundance in placentas with PE and malaria-relevant inflammation (n = 3) and without PE and inflammation (n = 19) was measured using immunofluorescence assay. Medians of arbitrary fluorescence units (AFU) are reported (gray bars). Protein abundance between 2 groups was compared using Mann-Whitney test. PE – parasitized erythrocytes in the placenta at the time of delivery.

When data were stratified by the presence of placental inflammation in malaria-infected samples (n = 3) against samples without infection and inflammation, a similar decrease in megalin and Dab2 abundance was observed (Fig. 2), with statistical significance for Dab2.

**Figure 3:**
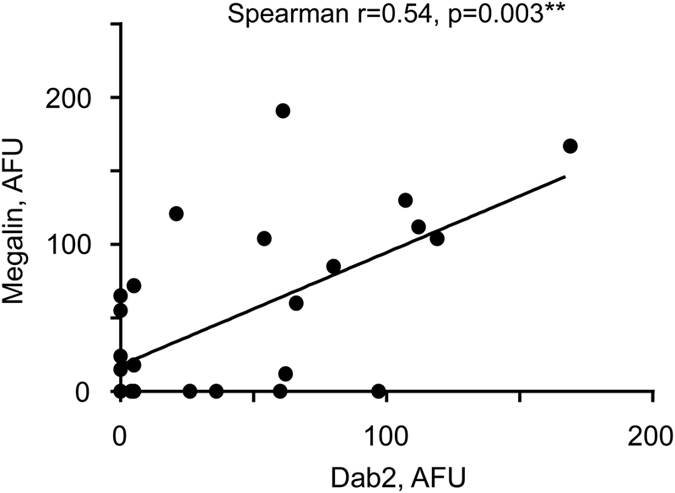
Correlation of megalin and Dab2 abundance in brush border area of syncytiotrophoblast. Correlation between amounts of Megalin and Dab2 was assessed using Spearman’s rank correlation coefficient (r). Megalin and Dab 2 abundance in all placentas (n = 28) was measured in arbitrary fluorescence units (AFU) using immunofluorescence assay as described in Methods. Six study participants (4 with PE and hemozoin, 1 with hemozoin only, and 1 without PE or hemozoin) did not have detectable megalin and Dab2. Line indicates linear regression, slope = 0.77 + 0.19, p = 0.0003. PE – parasitized erythrocytes in the placenta at the time of delivery. Hemozoin in the placenta at the time of delivery is indicative of past infection.

Also, irrespective of PM status of samples, statistically significant positive correlation of megalin and Dab2 expression in the brush border region was observed (Fig. 3). A similar correlation was observed when the entire syncytiotrophoblast was analyzed (data not shown).

### Expression of megalin and Dab2 is reduced in placentas with malaria parasites and low birth weight

**Figure 4:**
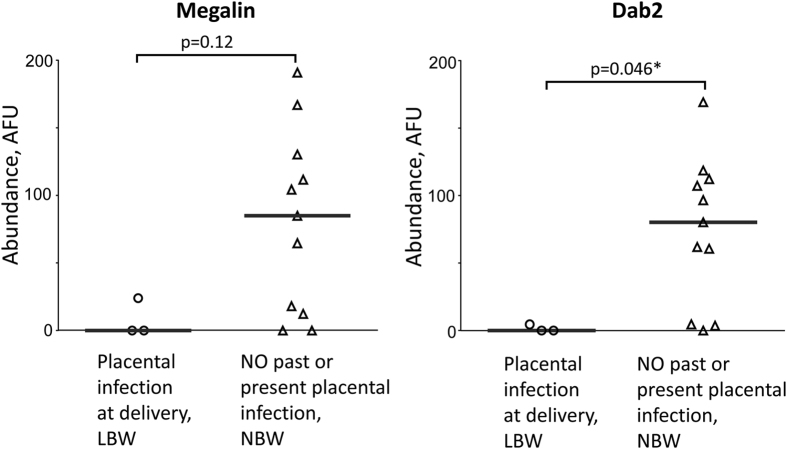
Megalin and Dab2 abundance is reduced in brush border area of syncytiotrophoblast in placentas with malarial infection and low birth weight. Megalin and Dab 2 abundance in placentas with PE and low birth weight (LBW) (n = 3) and without PE or past infection and normal birth weight (NBW) (n = 11) was measured using immunofluorescence assay. Medians of arbitrary fluorescence units (AFU) are reported (gray bars). Protein abundance between 2 groups was compared using Mann-Whitney test. PE – parasitized erythrocytes in the placenta at the time of delivery.

**Figure 5:**
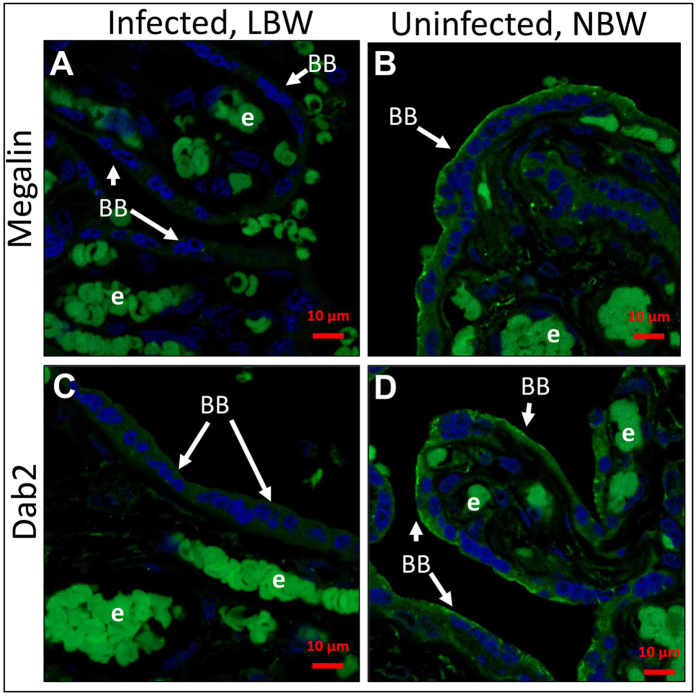
Expression of megalin and Dab2 in placentas from mothers with low birth weight babies is reduced. Megalin (**A,B**) and Dab2 (**C,D**) expression in syncytiotrophoblast of placentas with malaria infection and low birth weight (**A,C**) and without malaria infection and normal weight (**B,D**) was measured using immunofluorescence assay. Protein expression is shown in green, DAPI nuclear stain in blue. Pairs of pictures (**A–D**) were taken and processed under identical conditions. Bright cells inside of fetal vessels are auto-fluorescing erythrocytes (e). Brush border (BB) is indicated by arrows. LBW – low birth weight. NBW – normal birth weight.

Moderate correlation of birth weight with abundance of megalin and Dab2 in the brush border of the syncytiotrophoblast was identified, though below the level of statistical significance (Spearman correlation coefficients, Megalin: r = 0.35 [CI = −0.09981 to 0.6782], p = 0.11; Dab2: r = 0.23 [CI = −0.2242 to 0.6030, p = 0.3). When Dab2 expression levels were stratified by median Dab2 abundance, birth weight was 350 g greater in those with high (above median) versus low (below median) placental expression, although the difference was not statistically significant (p = 0.095) (a woman with stillbirth and no detectable Dab2 expression was excluded). Abundance of both megalin and Dab2 is substantially reduced in the brush border of malaria-infected placentas obtained from LBW deliveries (Fig. 4), with statistical significance for Dab2 (p = 0.046). Figure 5 illustrates comparative differences observed between two samples from infected placenta and LBW versus uninfected placenta and normal birth weight (NBW). Specifically, the brush border of syncytiotrophoblast demonstrates the low or absent levels of megalin and Dab2 in the LBW samples with active malaria infection.

## Discussion

PM-related changes in the maternofetal interface of the placenta are associated with LBW^6^. Several mechanisms contributing to LBW have been suggested, including dysregulated angiogenesis^9^,^10^,^37^, impaired growth hormone production^12^,^38^, and decreased nutrient transport^6^. In this study a novel role for megalin system in PM pathologies was explored, including PM-associated LBW. Results demonstrate that PM is associated with reduced syncytiotrophoblast abundance of megalin and Dab2 proteins, known to provide endocytosis and signaling pathways, which, in turn, may play a role in fetal growth restriction and low birth weight pathology.

Specifically, the study provides evidence that megalin and Dab 2 abundance is reduced in placentas with active infections (Fig. 1). Similar reduction (with statistical significance for Dab2) was also observed in infected placentas with inflammation (Fig. 2). As PM is often characterized by infiltration of immune cells, especially monocytes and macrophages, into intervillous space^39^, these results suggest that inflammation may affect megalin system expression in syncytiotrophoblast. The small number of samples with malaria-related inflammation (n = 3) limits statistical power in our analysis.

Further, amounts of megalin and Dab2 are positively correlated in the placental brush border (Fig. 3). It has been shown earlier that Dab2, as an intracellular ligand of megalin^25^, co-localizes with megalin in renal proximal tubules^40^. In addition, expression levels of both proteins are mutually dependent, where knockout of one of them reduces expression of the other one^40^. The results presented here expand this observation to placental tissue and indicate inter-relevance of megalin and Dab2 expression. Moreover, this underscores the importance of significant associations identified in this work, even found for one of these proteins, as reduction of its abundance may affect the function of the entire system.

While all samples with placental infection and LBW had low or undetectable megalin and Dab2 (3/3), there were 4/11 and 3/11 normal controls with NBW that also had low or undetectable levels of megalin and Dab2, respectively (Fig. 4). In addition, the reduction of megalin system proteins in the placenta correlated with the reduction in birth weight irrespective of PM status, but these data were not statistically significant. Though small sample size definitely limited the power of our analysis, it allows to speculate that such correlation or association might be the case not only during PM, but, potentially, in other diseases of pregnancy. Low levels of these proteins in a proportion of malaria-negative placentas may indicate that other factors might affect their abundance. For example, renal inflammatory processes such as those that occur in Heymann nephritis can lead to shedding of the megalin exodomain^34^; both megalin and its co-receptor cubilin were downregulated in gallbladder epithelium in gallstone patients at the mRNA and protein levels^41^; mice fed a cholesterol rich lithogenic diet showed a reduction in megalin mRNA expression in these cells^15^. Megalin expression might be responsive to treatment, for example, rosiglitazone significantly increased megalin mRNA expression in the gallbladder^15^. Similarly, multiple factors may contribute to the expression of megalin system proteins in uninfected placentas. Also, in spite of megalin importance, even megalin knock-out newborn mice can survive, though in low numbers (2%), indicating that some redundant mechanism(s) may compensate for megalin system insufficiency during fetal development which allows for survival^20^,^21^.

Nevertheless, categorical analysis of protein abundance data stratified by LBW and placental infection revealed statistically significant reduction for Dab2 abundance (and very similar trend for megalin) compared to non-infected placental samples with NBW (Fig. 4). This result suggests that reduction in abundance of megalin transport/signaling system proteins in PM may contribute to the pathology of LBW. Below is a discussion of the potential mechanisms of pathological changes in pregnancy in relevance to the disturbance of the megalin system in placenta, which was observed in this work during PM.

Maternal cholesterol is essential for fetal growth^42^. It has been shown that fetal growth restriction is associated with alterations in placental lipoprotein receptors (LDL, LRP1) and maternal lipoprotein composition^43^. Megalin is involved in transport of the same lipoparticles, plus it interacts and transports additional types of Apo-lipoproteins and might substantially contribute to cholesterol transport, necessary for fetal growth.

Megalin also plays an important role in vitamin homeostasis by re-absorbing various *vitamins* in complexes with their carrier proteins in kidneys^19^,^44^. Because vitamins for the developing fetus can only be supplied from maternal blood, megalin may play a similar function in placenta supplying retinol^22^, cobalamins^45^, folate, and vitamin B12^45^, as well as vitamin D, which is important for regulation of calcium homeostasis^46^. Moreover, deficiency of vitamin D in pregnancy may increase the risk of preterm delivery and fetal growth restriction^47^.

Further, megalin co-expresses on the syncytiotrophoblast surface with several receptors specialized for interactions with hormones/growth factors (that are also megalin ligands) including insulin-like growth factor-1 (IGF-1), parathyroid hormone and epidermal growth factor^5^, which play important roles in the regulation of fetal growth throughout pregnancy^48^. Thyroid hormones (TH) are involved in placenta villous development and free TH (T4) levels are associated with birth weight^49^. In maternal blood, T4 is almost completely bound to transthyretin, a ligand for megalin^50^ that is secreted to maternal circulation and re-absorbed by trophoblasts^51^, and therefore may maintain T4 homeostasis. As mentioned above, PM was associated with disturbance of the IGF axis, with significant changes in IGF-1 but not IGF-2, and no detectable changes in mRNA levels for IGF-1 receptor^12^. Since IGF-1 is also a ligand for megalin and modulation of the megalin system may affect levels of IGF-1^52^, megalin reduction may contribute to the effects of PM on IGF-1 level changes^12^.

How does PM affect the megalin system? One potential mechanism might be that sequestration of PE through surface-expressed PfEMP1 parasite adhesins may cross-link the corresponding host adhesion receptor, chondroitin sulfate A^1^, on the syncytiotrophoblast surface. This clustering of the surface receptors may directly affect multiple membrane processes including megalin-mediated endocytic and/or signaling pathways, as was the case in other studies on the effects of cross-linking of other cell surface receptors^53^. Infiltrating macrophages during PM may directly damage the syncytiotrophoblast surface, linking placental inflammation and megalin system abundance (Fig. 2) and/or function.

TGFβ negatively regulates endocytosis of albumin through megalin and cubilin^54^. During placental malaria TGF-beta concentration in placenta is significantly increased^55^. Hence, systemic regulation of placental megalin expression in malaria is possible and may lead to reduced endocytosis in infected placentas.

This study is limited by the number of samples investigated. A logical continuation would be a similar study with larger sample size and samples obtained from various geographical areas, taking into account a larger set of variables, including timing of malaria infection during pregnancy and nutritional status. Further, effects of PE adhesion to cytotrophoblast primary cells or cell lines, like BeWo cells, on megalin system functioning in the absence and presence of monocytes/macrophages may test our hypothesis *in vitro*. These studies may contribute to development of therapy alleviating pathological effects of megalin system dysfunction, for example by affecting transcription of relevant genes, and/or by supplementation of nutrients and ligands.

In summary, it is likely megalin is involved in fetal growth via a complex network of regulatory activities participating in endocrine, paracrine, and autocrine signaling by interacting with and internalizing various ligands of maternal and fetal origin. We propose that dysregulation of these networks in the placenta affects development of the fetus. To our knowledge, this is the first report linking the abundance of placental megalin system proteins with the birth weight of newborn babies and associating PM infection with changes in abundance of megalin system proteins.

## Methods

### Ethics Statement

Written informed consent was provided and specimens were collected under approval by the Uganda National Committee for Science and Technology (#HS207). The use of coded specimens was approved and deemed not human subjects research by the University of Washington Human Subjects Division (#35425) and by the Florida Atlantic University Institutional Review Board (#801504). All experiments were performed in accordance with the relevant guidelines and regulations.

### Placental samples

Specimens were from a cohort of a nested randomized control trial of artemether-lumefantrine versus quinine to treat malaria in pregnancy conducted in 2006–2009 in Mbarara, Uganda, as previously described^35^. Briefly, the randomized controlled trial inclusion criteria included viable pregnancy at gestational age of 13 weeks or later and positive malaria blood smear by microscopy; exclusion criteria included *P. falciparum* parasitemia greater than 250 × 10^3^/μL, severe anemia (hemoglobin < 7 g/dL), or severe or complicated malaria needing parenteral treatment. As previously described^56^, placental biopsies were fixed and stored in neutral buffered formalin, routinely processed and scored for the presence or absence of parasitized erythrocytes (PE + or PE−) and hemozoin in fibrin^57^, which were semi-quantitatively scored together with the presence of intervillous inflammatory infiltrates^58^. Placentas positive for parasitized erythrocytes (PE) by histology were referred as malaria-infected. To select samples for this study (n = 28) all available samples that had parasites identified by histology (n = 8) were matched to sequential controls that had no identifiable placental malaria-related changes on histopathology (n = 17), maintaining similarities in parity (Supplementary Figure S2). These control samples included available placental specimens from the larger cohort of women, who were screened negative for malaria by blood smear at weekly visits and not included in the randomized controlled trial^36^ (samples 23–28 in Supplementary Table S1). In addition, a subset of women with a relatively large amount of hemozoin in fibrin but no parasites (heavy past infections) were also available and were included (n = 3).

For the immunofluorescence assays, sections of 6 μm were prepared from paraffin embedded blocks of formalin-fixed placentas obtained from these women. Relevant clinical parameters of placenta samples are described in Supplementary Table S1.

### Antibodies

Primary antibodies used were monospecific purified rabbit antibodies, raised against megalin C-terminal 17-mer peptide (2 independently purified preparations) and Dab2 phosphotyrosine interaction domain, which were described and characterized previously^25^, and commercial anti-Dab2 antibody H-110 (# sc-13982, Santa Cruz Biotechnology, CA). Secondary antibodies used were Alexa Fluor 594-labeled polyclonal Donkey anti-Rabbit IgG (#711-585-152, Jackson Immunoresearch, West Grove, PA).

### Measurement of megalin and Dab2 abundance by indirect fluorescence microscopy

The paraffin embedded tissue samples were rehydrated by a series (3 min each) of graded xylene/ethanol washes (2 times with 100% xylene, once with 1:1 xylene/ethanol, once sequentially with 100%, 95%, 70% and 50% ethanol in deionized water), followed by a final wash in phosphate buffered saline (PBS) for 5 min. The rehydrated slides were then heated in Tris-EDTA buffer (10 mM Tris Base, 1 mM EDTA solution, 0.05% Tween-20, pH 9.0) and microwaved to boiling at full power and then maintained at 50% power for 8 minutes and cooled in PBS^59^. Sections were then pre-incubated with PBS, 1% BSA and 0.25% Triton-X for 30 minutes, incubated with primary antibody (Dab2 commercial 1:100; Dab2-PID 1:100; Megalin C-terminal 17-mer peptide 1:50) in the presence of 2.5% non-immune donkey serum (#017-000-121, Jackson Immunoresearch, West Grove, PA, USA), either for one hour at room temperature or overnight at 4 °C in a humidified chamber and washed with PBS for 5 minutes 3 times. Slides were then incubated with secondary antibody in the presence of non-immune donkey IgG (# 017-000-002, 1:100, Jackson Immunoresearch, West Grove, PA, USA) for one hour at room temperature and counterstained with DAPI (5 μg/mL, Sigma) for 3 minutes. After three washes with PBS for 5 minutes, slides were processed with mounting medium (Vectashield H-1000, Vector Laboratories, Burlingame, CA, USA) and coverslip applied. Negative controls were processed without primary antibody or with antibodies purified from the pre-immunized animal serum on the same column as monospecific antibodies. Both methods produced similar background levels (data not shown). For the staining, to provide maximally identical conditions, each placental section was divided in sub-sections processed with all primary antibodies, and then incubated with secondary antibody simultaneously. As a positive control for the procedures used, we stained fresh-frozen mouse kidney 6 μm sections as described above and obtained strong staining of the proximal tubule cells with anti-megalin and anti-Dab 2 antibodies, as described previously^40^,^60^ (data not shown).

Slides were imaged using a confocal microscope (Zeiss LSM 700 equipped with Plan-Apochromat 63x/1.40 Oil DIC M27 objective) and processed using Zen 2012 Blue software (Zeiss, Jena, Germany). Images were captured using identical conditions to allow for quantification of signal strength. Collected images of syncytiotrophoblast were selected for the brush border only (~1 μm from the surface to inside of the cell) or the entire syncytiotrophoblast (up to ~10 μm in depth and excluding nuclei). The process of selection is illustrated in Supplementary Figure S3: selected region A indicates brush border area, and region A+B indicates entire syncytiotrophoblast. In each placental section at least 3 non-overlapping areas encompassing syncytiotrophoblast 50 μm–100 μm along the surface were selected for the highest density of signal (in arbitrary fluorescence units, AFU) using visual appearance and density-measuring feature of the Zen 2012 Blue software, measured as described above, and averaged for each placental sample. If staining was similar throughout the section, 3 random areas were selected. Negative control AFU were then subtracted from the experimental AFU to obtain final AFU for each sample used in the analysis. All slides were processed by the same individual, blindly.

### Statistical analyses

Statistical analyses were performed using GraphPad Prism 6 (La Jolla, California, US) statistical software using non-parametric statistics: Mann-Whitney tests (two-tail) for group differences and Spearman tests for analyses of correlations. P value of <0.05 was considered significant. Qualitative level of significance indicated by stars: *p < 0.05, **p < 0.01, ***p < 0.001.

## Additional Information

**How to cite this article**: Lybbert, J. *et al.* Abundance of megalin and Dab2 is reduced in syncytiotrophoblast during placental malaria, which may contribute to low birth weight. *Sci. Rep.* **6**, 24508; doi: 10.1038/srep24508 (2016).

## Supplementary Material

Supplementary Information

## Figures and Tables

**Table 1:** Clinical characteristics of 28 Ugandan pregnant women at delivery.

	Mothers without placental infection (PE−)	Mothers with placental infection (PE + )	P value^a^
Number of women	20	8	
Parity median [interquartile range]; n	1 [1.25]; 16	1 [1]; 6	0.88
Hemoglobin level [g/dL] mean (SD); n	11.4 (2.3); 16	10.5 (2.1); 5	0.37
Estimated gestational age [weeks] mean (SD); n	39 (2); 15	38 (4); 5	0.88
Birthweight [g] mean (SD); n	2916 (513); 16	2942 (476); 5	0.89
Low birth weight n (%)	4/16 (25%)	2/6 (33%)	
Stillbirth n (%)^b^	0/16 (0%)	1/6 (17%)	

^a^Mann-Whitney test was used to calculate all p values.

^b^This sample was included in categorical analysis as low birth weight.
