# Supplementary material for: Abundance of megalin and Dab2 is reduced in syncytiotrophoblast during placental malaria, which may contribute to low birth weight
Source: Sci Rep. 2016 Apr 13;6:24508. doi: 10.1038/srep24508 (PMC4829923; doi:10.1038/srep24508)
Supplement: Supplementary Information [file srep24508-s1.pdf]

Supplementary Information

Abundance of megalin and Dab2 is reduced in syncytiotrophoblast during placental malaria, which may contribute to low birth weight

Jared Lybbert[1], Justin Gullingsrud[2], Olga Chesnokov[1], Eleanor Turyakira[3], Mehul Dhorda[4,5], Philippe J. Guerin[4,5], Patrice Piola[5], Atis Muehlenbachs[6], Andrew V. Oleinikov[1,2,*]

Supplementary Table S1. Clinical information on placental sections and corresponding babies and women. Parasite – parasites found in placenta at delivery; hemozoin – extracellular hemozoin in fibrin found in placenta indicating previous (resolved) placental infection; inflammation – inflammatory cells found in placenta; birth weight – measured at delivery; parity indicates previous pregnancies. Abbreviations: y – yes, n – no, n/a – not available, Hgb – hemoglobin, AL – artemether-lumefantrine, Q – quinine, LBW – low birth weight

Sample #	Parasite	Hemozoin	Inflammation	Birth weight, g	Parity	Hgb	Study Arm	Notes
1	n	n	n	3180	1	11.7	AL	
2	n	n	n	2490	0	11.9	AL	LBW
3	n	y	n	2310	1	12.0	AL	LBW
4	n	n	n	3470	0	9.7	Q	
5	n	n	n	2040	0	11.1	Q	LBW
6	y	y	n	2430	0	8.8	AL	LBW
7	y	y	n	2430	2	11.0	Q	LBW
8	y	y	n	3300	0	11.6	AL	
9	n	n	n	3050	1	12.8	AL	
10	y	y	n	3150	1	13.0	AL	
11	n	n	n	3810	0	11.3	Q	
12	n	n	n	2880	1	9.8	Q	
13	n	n	n	3500	0	16.0	Q	
14	n	n	n	2000	3	5.8	Q	LBW
15	y	n	n	3400	1	8.0	Q	
16	y	y	y	n/a	1	n/a	AL	Stillbirth*
17	n	n	n	2900	2	10.7	Q	
18	n	n	n	2880	0	10.2	Q	
19	n	n	y	2770	1	12.3	Q	
20	n	y	n	2860	0	10.1	AL	
21	n	n	n	3240	2+	13.8	Q	
22	n	n	n	3280	1	13.3	Q	
23	n	n	n	n/a	n/a	n/a		
24	y	y	y	n/a	n/a	n/a		
25	y	y	y	n/a	n/a	n/a		
26	n	y	n	n/a	n/a	n/a		
27	n	n	n	n/a	n/a	n/a		
28	n	n	n	n/a	n/a	n/a		

*This sample was included in categorical analysis as low birth weight

Supplementary Figure Legends

Supplementary Figure S1. Strong correlation of protein abundance measurements in sections of placental samples obtained in independent blind experiments using two different antibody preparations. A, megalin measurements. B, Dab2 measurements.

Supplementary Figure S2. Parity is not a confounding factor in association of placental infection with reduced abundance of megalin and Dab2. Samples with data on parity (n=22, Supplementary Table 1) were used for this analysis. Red bars, medians. PE, infected erythrocytes in placenta at the time of delivery.

Supplementary Figure S3. **Quantification of protein abundance in syncytiotrophoblast.** Selected region A indicates brush border area, and region A+B indicates entire syncytiotrophoblast. Three areas like this per section were selected and used for measurements with each antibody. Results were averaged.

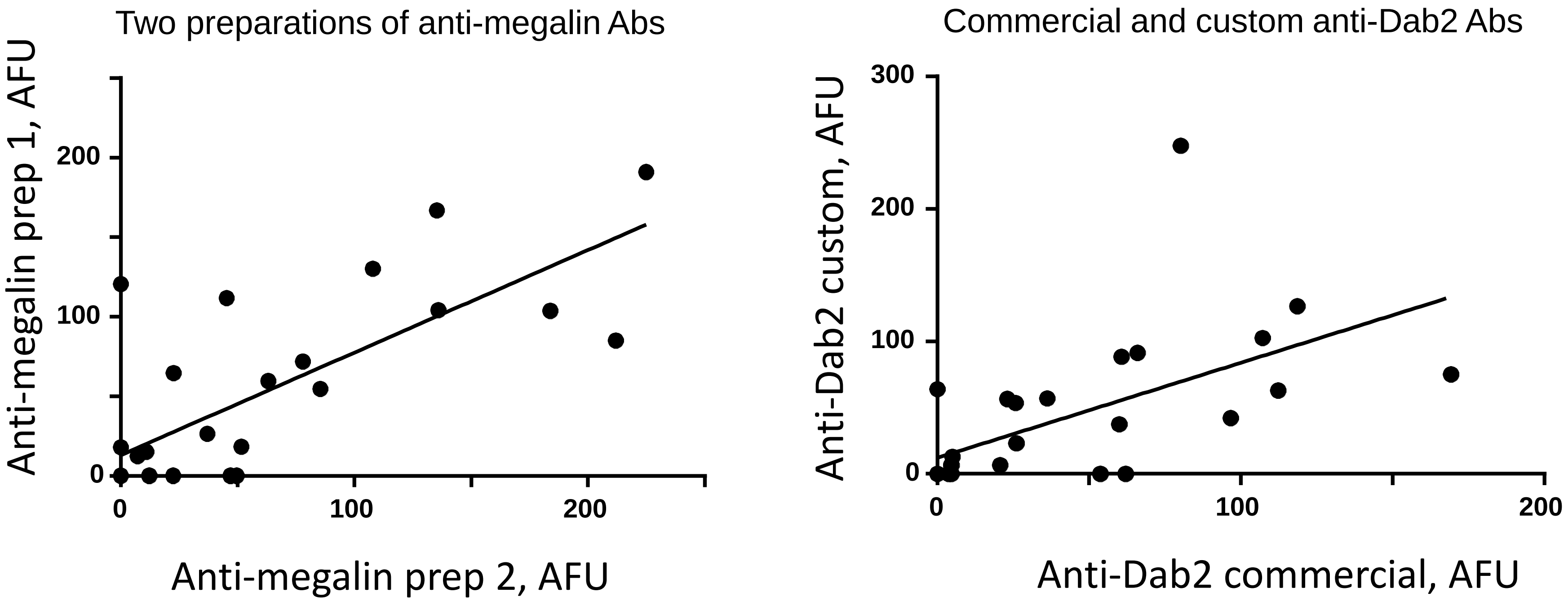

Spearman correlation r=0.69, p<0.0001
Line shows linear regression

Spearman correlation r=0.73, p<0.0001
Line shows linear regression

Supplementary Figure S1. Strong correlation of protein abundance measurements in sections of placental samples obtained in independent blind experiments using two different antibody preparations. A, megalin measurements. B, Dab2 measurements.

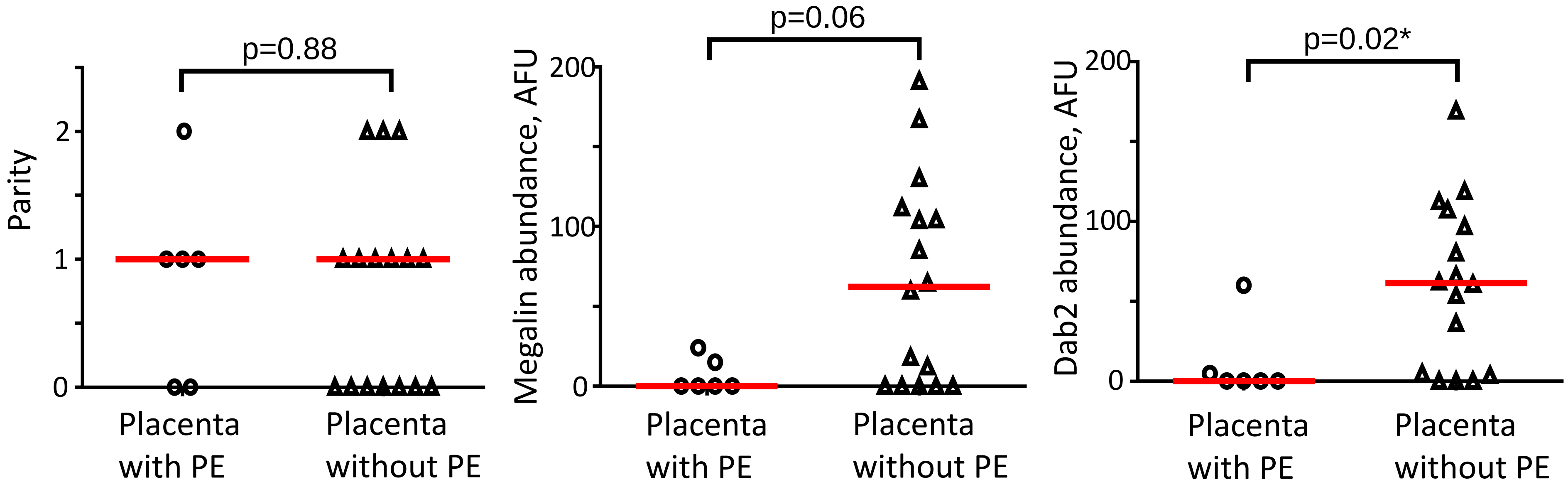

Supplementary Figure S2. Parity is not a confounding factor in association of placental infection with reduced abundance of megalin and Dab2. Samples with data on parity (n=22, Supplementary Table 1) were used for this analysis. Red bars, medians. PE, infected erythrocytes in placenta at the time of delivery.

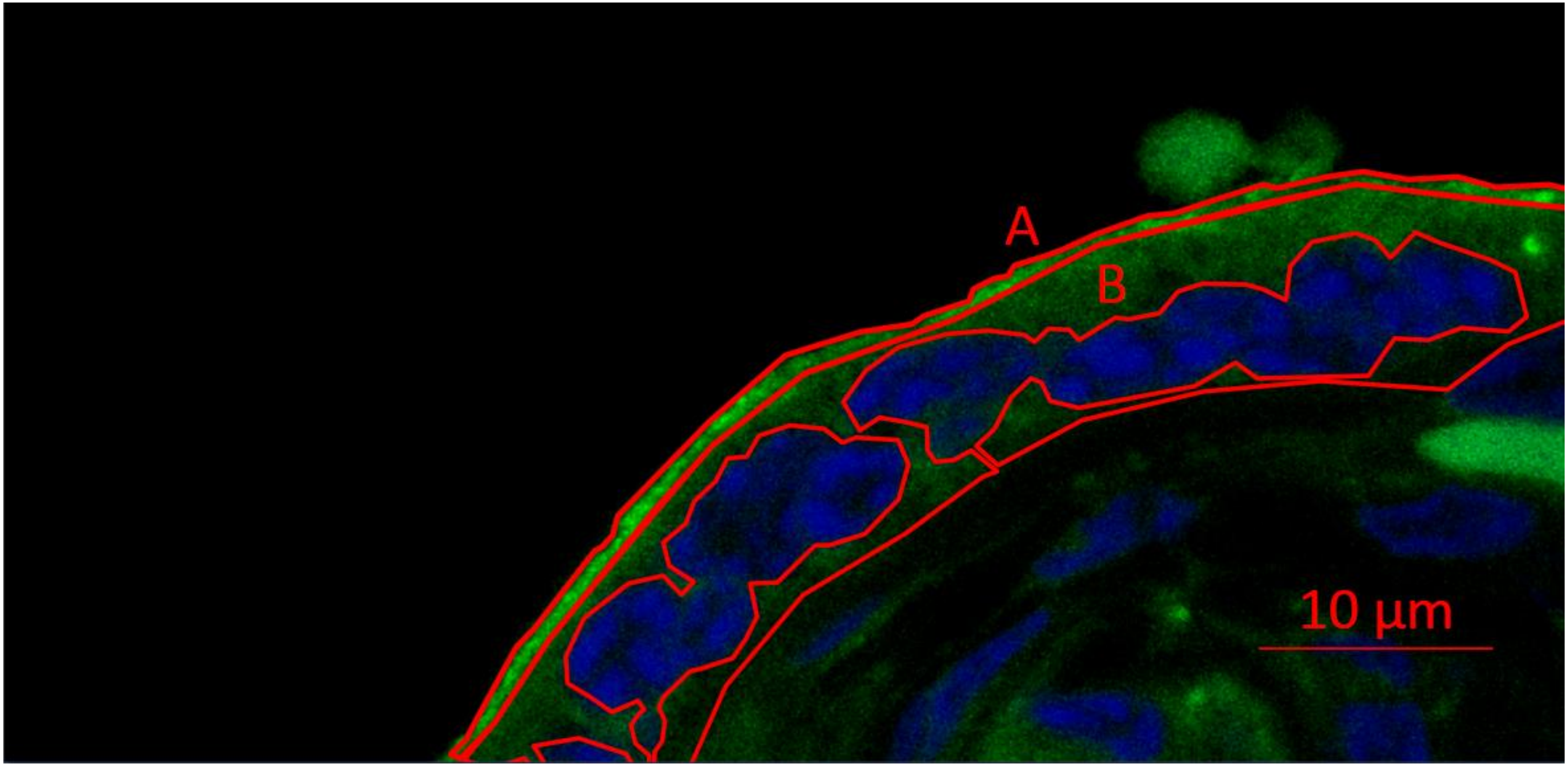

Supplementary Figure S3. Quantification of protein abundance in syncytiotrophoblast. Selected region A indicates brush border area, and region A+B indicates entire syncytiotrophoblast. Three areas like this per section were selected and used for measurements with each antibody. Results were averaged.
